# The stagnation of child anaemia (6–23 months) in Tanzania from 2004 to 2022: a missed opportunity during the ‘first 1000 days’

**DOI:** 10.7189/jogh.16.04033

**Published:** 2026-02-13

**Authors:** Yuwen Wang, Bruno Sunguya, Muzi Na, Mayassa Salum Ally, Jiayan Huang

**Affiliations:** 1 School of Public Health, Global Health Institute, Fudan University, Shanghai, China; 2 School of Public Health and Social Sciences, Muhimbili University of Health and Allied Sciences, Dar es Salaam, Tanzania; 3 Department of Nutritional Sciences, The Pennsylvania State University, University Park, Pennsylvania, USA; 4 Zanzibar National Health Research Institute, Zanzibar, Tanzania; 5 Institute of Belt and Road & Global Governance, Fudan University, Shanghai, China

## Abstract

**Background:**

Child anaemia remains a major public health concern in low- and middle-income countries, particularly among children aged <2 years. Given Tanzania’s slow progress towards addressing this challenge, despite the implementation of targeted national strategies, we aimed to examine the prevalence and associated factors of anaemia in children aged <2 years.

**Methods:**

This cross-sectional study was based on secondary data from the Tanzania Demographic and Health Surveys (2004–2005, 2010, 2015–2016, and 2022). We applied individual sampling weights to ensure national representativeness and used descriptive analyses to estimate the prevalence of child anaemia, both overall and in specific age group distributions (6–23 and 24–59 months), by severity. Then, employed geographic information system mapping to visualise the regional anaemia prevalence among children aged <2 years. Finally, we conducted a multivariable logistic regression analysis to identify risk factors related to child anaemia and sensitivity analyses to check the robustness of our findings.

**Results:**

We included 25 590 children aged <5 years with available haemoglobin data, of whom 10 166 were aged 6–23 months. In 2022, 59.9% of children aged <5 years and 73.1% of those aged 6–23 months were anaemic. Using spatial analysis, we found that 16 of 31 regions experienced increased prevalence of anaemia, while the remaining 15 saw slight to moderate declines since 2015. In the multivariable analysis, being male (adjusted odds ratio (aOR) = 1.380; 95% confidence interval (CI) = 1.133–1.681), having low birth weight (aOR = 1.713; 95% CI = 1.076–2.727), being underweight (aOR = 1.438; 95% CI = 1.032–2.005), and not having health insurance (aOR = 1.768; 95% CI = 1.247–2.508) were factors significantly associated with increased risk of anaemia.

**Conclusion:**

s Child anaemia in Tanzania has stagnated between 2015–2022, failing to meet national targets. The burden has remained disproportionately high among low birth weight children and those aged <2 years, with those residing in coastal regions and high food production areas being especially vulnerable. Targeted interventions during the first 1000 days of life should be prioritised to break the intergenerational cycle of anaemia.

Child anaemia is a global public health issue [1,2], with the World Health Organization (WHO) estimating that approximately 40% of children aged 6–59 months had anaemia in 2019 [3]. While this global burden of this condition has improved since 2000 [4,5], including in sub-Saharan Africa, which experienced a significant decline in cases of severe anaemia [6], it remains significantly high in low- and middle-income countries (LMICs), with some variations. Tanzania, for example, had a prevalence of 59% among children aged <5 years in 2019 [7]. In fact, the country had a high (>55%) prevalence of child anaemia for almost 20 years, which has been slow to decline [8], with the WHO estimating it decreased by no more than 3% over the past five years (2015–2019) [3].

To address widespread nutritional challenges, Tanzania introduced the National Multisectoral Nutrition Action Plan (NMNAP) in 2016, aiming to reduce the prevalence of child anaemia from the 2015 baseline of 58% to 30% by 2021 [8]. The second phase (NMNAP II), launched in 2021, aligns with the Sustainable Development Goals, particularly the goal of eliminating hunger and all forms of malnutrition by 2030. However, recent evidence suggests that achieving the WHO’s 2030 targets for reducing child anaemia will be highly challenging for LMICs [9], while most national-level studies in Tanzania were conducted before 2020 [8,10]. To estimate the country’s progress towards these targets, it is necessary to assess whether child anaemia in Tanzania has declined to the expected level, especially following the economic and societal shock stemming from the COVID-19 pandemic, and to identify the persistent challenges hindering further reductions in the disease burden.

Research has identified several core factors related to child anaemia in Tanzania [11,12]. The condition is predominantly nutritional and manifests itself in the form of iron deficiency anaemia, which is a particularly high burden among children aged <2 years [2]. Other mechanisms include nutritional interactions, such as nutrient loss, impaired absorption, and inadequate iron stores at birth [13]. Beyond these physiological causes, studies have found sociodemographic factors to influence child anaemia in Tanzania [14], such as age, gender, and diet in low- and middle-income areas, or household factors in general, especially maternal factors and wealth index [10,15]. Moreover, significant regional disparities exist in the prevalence of child anaemia across the country, partly due to differences in infectious disease burden, shaped by regional climate conditions and sanitation infrastructure [16,17]. Such regional differences in economic activities influence dietary patterns and individuals’ socioeconomic status, further contributing to the heterogeneity in anaemia prevalence.

However, there is a notable lack of evidence on children aged <2 years, specifically. Together with maternal gestation, this critical development period constitutes the ‘the first 1000 days’ of their life [18], when children are most susceptible to the adverse consequences of anaemia [18]. As highlighted by the United Nations Children’s Fund (UNICEF), adequate nutrition and healthcare during the first 1000 days can significantly impact a child’s brain development, immunity, and growth, ultimately affecting their health through adulthood and into society [19].

In this study, we aimed to describe the prevalence and severity of anaemia among children aged 6–23 months and to identify individual- and household-level factors associated with anaemia status among children aged <2 years, using data from the Tanzanian Demographic and Health Survey (TDHS). The expected outcomes could provide insights into the situation of child anaemia in Tanzania over the past decade and offer evidence-based recommendations and support.

## METHODS

### Study design and data sources

This cross-sectional study was based on secondary data from the TDHS conducted in 2004–2005, 2010, 2015–2016, and 2022. The TDHS is undertaken every four years by the Tanzania National Bureau of Statistics in collaboration with the Ministry of Health in Tanzania mainland and Zanzibar, with technical and financial support from the United States Agency for International Development. It uses a two-stage stratified cluster sampling design where, in the first stage, enumeration areas are selected using probability proportional to size sampling. In the second stage, a fixed or variable number of households is systematically chosen with equal probability within each selected enumeration area. Eligible individuals within these households are then interviewed individually. The TDHS primarily collects data on the health and well-being of children aged <5 years, health service provision, and other social determinants of health from women in the selected households.

In this study, we weighted the TDHS data using individual sampling weights to ensure national representativeness. In addition, we accounted for the stratification and clustering inherent in the TDHS sampling design to obtain valid population-based estimates.

### Variables and measurements

Our primary outcome variable was child anaemia, which was assessed by measuring haemoglobin concentration. In the TDHS survey, testing was done using a HemoCue photometer (HemoCue AB, Ängelholm, Sweden) from a drop of capillary blood obtained by finger or heel prick. The devices, mainly haemoglobin 201 or 201+ models, were calibrated daily following the manufacturer’s instructions. Standardised field protocols, technician training, and quality control procedures ensured data comparability across surveys.

We adjusted haemoglobin values for altitude, applying corrections for survey clusters at elevations above 1000 m to account for reduced barometric pressure. Anaemia among children aged 6–59 months in TDHS was classified according to the WHO criteria, operationally defined as a haemoglobin concentration of <11 g/dL [13], into three severity categories: mild (haemoglobin 10–11 g/dL), moderate (haemoglobin 7–9.9 g/dL), and severe (haemoglobin <7 g/dL).

We included individual and household factors identified in the literature as associated with child anaemia [20] as independent variables. Individual characteristics included the child’s age (in months), sex, dietary diversity score, stunting, underweight, and birth weight. We harmonised dietary diversity across waves using the WHO 2021 definition, which classifies five or more distinct food groups as adequate. Due to limitations concerning data availability, we included this variable only for the two most recent TDHS. Following the DHS Statistics guide, we coded missing data as non-consumption of that food group [21]. We defined stunting as a height-for-age Z-score more than two standard deviations (SD) below the median of the WHO Child Growth Standards. We employed the weight-for-age Z-score (WAZ) to categorise the weight status of children aged 0–59 months into the following categories: underweight (WAZ<−2 SD), normal weight (−2 SD≤WAZ≤+2 SD), and overweight (WAZ>+2 SD). We classified birth as low (<2500 g), normal (2500–4000 g), or high (≥4000 g). Household characteristics included residence (urban *vs.* rural), health insurance (mother), the weighted wealth index, total number of household members, the number of children under five years of age within the household, the mother’s age at first childbirth, marital status, household insecticide-treated net use, and the highest level of education attained ranging from no education, primary, secondary, to higher level of education. We computed the weighted wealth index using principal component analysis and factor analysis of household asset ownership, aggregating factor loadings as sample weights to yield the weighted wealth index. We defined household insecticide-treated net use as whether a child aged <5 years slept under an insecticide-treated net the night before the survey.

Additionally, we reported covariates with remaining missing values after data preparation, including birth weight, stunting, and weight (Table S1 in the **Online Supplementary Document**).

### Data analysis

We conducted descriptive analyses to examine the overall prevalence of child anaemia and its distribution by severity level across two age subgroups: 6–23 months and 24–59 months. We used the Pearson χ^2^ test to examine differences in anaemia associated with demographic and household characteristics of children aged <2 years that are hypothesised to be associated with child anaemia.

We employed geographic information system mapping to visualise the spatial distribution of child anaemia prevalence across Tanzania’s districts and to compare these patterns between the 2015–2016 and 2022 surveys. To ensure comparability considering administrative boundary revisions, we restricted the analysis to the 2015–2016 and 2022 data sets. Songwe was separated from Mbeya as an independent region during this period. Accordingly, we calculated the change in anaemia prevalence by comparing the prevalence in each 2022 region, Mbeya and Songwe, with that in Mbeya in 2015–2016.

To quantify the net change in anaemia prevalence between the two most recent surveys and to explore factors associated with child anaemia among children aged <2, we performed a multivariable logistic regression analysis using pooled data from 2015–2016 and 2022. Before doing so, we first tested multicollinearity using the generalised variance inflation factor. All covariates passed the test and were retained in the model (Table S2 in the **Online Supplementary Document**). We included survey year as the independent variable and anaemia status (1 = anaemic; 0 = non-anaemic) as the dependent variable. We adjusted the model for demographic variables, household characteristics, and other health-related factors.

To assess the robustness of our findings, we conducted three sensitivity analyses. Given that the missingness rate in the pooled birth weight data exceeded 10%, we applied multiple imputation using both covariates and outcomes to mitigate potential bias from complete-case analysis. We also re-ran the regression model to evaluate the possibility of over-adjustment, excluding underweight. Finally, we applied alternative cut-offs for moderate anaemia to examine whether the results were sensitive to the definition of anaemia.

We conducted the analyses of these data in three steps, and we used Stata, version 17.0 (StataCorp, College Station, Texas, USA) and *R*, version 4.4.3 (R Core Team, Vienna, Austria). We analysed data using descriptive, geographic information system mapping, and regression analysis methods.

### Ethics

Ethical approval was not required for this secondary analysis. The original data collection had obtained national ethical clearance and informed consent, and permission to use the data was sought through the DHS website.

## RESULTS

### The prevalence of child anaemia in Tanzania

**Figure 1:**
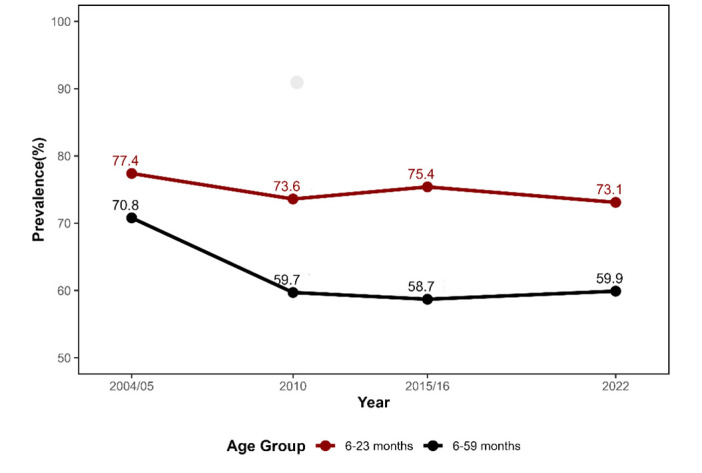
Trends in the prevalence of child anaemia between 2004 and 2022.

A total of 25 590 children aged <5 years were included after retaining all data that included haemoglobin measurements, with 7361, 6067, 7828, and 4334 participants in the survey waves 2004–2005, 2010, 2015–2016, and 2022, respectively. The smaller sample in 2022 reflects that, due to external factors such as the COVID-19 pandemic and cost-saving measures, only a 50% random subsample of households was selected for biological testing. There were 3319, 2215, 3034, and 1598 children aged 6–23 in the 2004–2005, 2010, 2015–2016, and 2022 waves, respectively. The prevalence of anaemia in the three TDHSs after 2004–05 remained relatively unchanged in Tanzania; it was estimated to be 59.9% in 2022, presenting an increase of 1.2 compared with 2015 levels (**Figure 1**).

**Figure 2:**
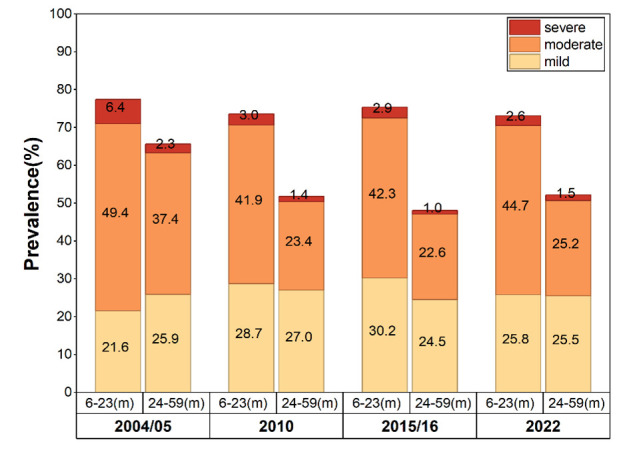
Child anaemia prevalence by severity between two age groups between 2004 and 2022.

The prevalence of anaemia among children aged 6–23 months, specifically, has consistently remained above 70.0% across all TDHS, with estimates placing it at 73.1% in 2022, representing little improvement over the 77.4% reported in the 2004–05 TDHS (**Figure 1**). The prevalence of severe and moderate anaemia in this group was 2.6% and 44.7%, respectively. In the 24–59-month-old age group, the prevalences of severe and moderate anaemia were 1.5% and 25.2%, respectively. The prevalence of mild anaemia was comparable between the two age groups, at 25.8% and 25.5%. We observed similar age-related differences in other survey years (**Figure 2**).

**Figure 3:**
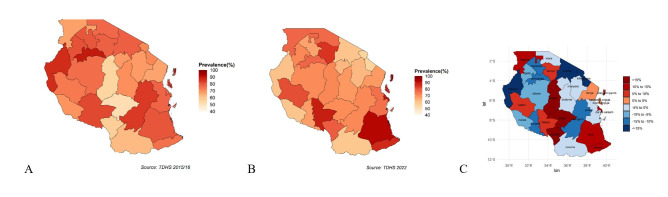
The GIS mapping of the regional burdens of child anaemia (6–23 months) in 2015–2016 and in 2022. **Panel A.** Regional prevalence of child anaemia in Tanzania, 2015–2016. **Panel B.** Regional prevalence of child anaemia in Tanzania, 2022. **Panel C.** Changes in regional child anaemia prevalence between 2015 and 2016 and in 2022 in Tanzania. Negative values indicate a decrease, positive values indicate an increase.

The prevalence of anaemia among children aged <2 years varies markedly across regions. In the 2022 TDHS, 27 of Tanzania’s 31 regions registered anaemia prevalence exceeding 60.0%, with Kaskazini Pemba exhibiting the highest values at 92.7% (**Figure** **3**, Panel B). In 2015–2016, 27 of 30 regions reported anaemia prevalence above 60.0% (**Figure 3**, Panel A). Some of the extreme values may be influenced by limited sample sizes at the regional level.

Comparing 2022 with 2015–2016, 16 regions faced worsening trends in anaemia prevalence in all 31 regions (using the 2022 administrative boundaries), particularly in the Lake Zone and the Southern Highlands. Among these 16 regions, three regions showed increases of more than 15 percentage points: Iringa (18.8%), Singida (16.5%), and Njombe (22.1%) (**Figure 3**, Panel C). In contrast, the remaining 15 regions recorded declines in anaemia prevalence, with Kigoma showing the greatest reduction of 25.4 %.

### The characteristics of child anaemia in Tanzania

Across all survey years, boys aged 6–23 months consistently exhibited a higher prevalence of anaemia compared to girls. For instance, in 2022, the anaemia prevalence among girls was 69.9%, while that among boys reached 76.4% (*P* = 0.016). Regarding maternal insurance coverage, we did not observe a statistically significant difference across the first two survey rounds. However, in 2015–2016, children whose mothers were uninsured had a significantly higher prevalence of anaemia compared to those whose mothers had insurance (74.1% *vs.* 64.9%; *P* = 0.001). The same pattern persisted in 2022, with anaemia prevalence of 74.1% *vs.* 48.8%, respectively (*P* < 0.001). Furthermore, some subgroups consistently experienced extremely high anaemia prevalence. Children with low birth weight showed persistently elevated anaemia rates across all years: 81.7% (2004–2005), 86.0% (2010), 84.0% (2015–2016), and 82.6% (2022). Similarly, anaemia prevalence among underweight children remained alarmingly high, reaching 80.4% in 2015–2016 and remaining at 80.5% in 2022 (**Table 1**).

**Table 1:** Individual and household characteristics in relation to the burden of child anaemia (6–23 months) in Tanzania

	Year											
	**2004–2005**			**2010**			**2015–2016**			**2022**		
**Variables**	**Anaemia cases, n**	**Total, n (%)**	***P*-value**	**Anaemia cases, n**	**Total, n (%)**	***P*-value**	**Anaemia cases, n**	**Total, n (%)**	***P*-value**	**Anaemia cases, n**	**Total, n (%)**	***P*-value**
Age						0.000*						0.332
*6–11*	1250	1721 (72.6)	<0.001	589	736 (80.0)		773	976 (79.2)	0.004	389	518 (73.1)	
*12–23*	1320	1598 (82.6)		1041	1479 (70.4)		1515	2058 (73.6)		779	1080 (72.1)	
Sex						0.005						0.016
*Female*	1256	1673 (75.0)	0.005	807	1144 (70.5)		1084	1493 (72.6)	0.002	546	784 (69.9)	
*Male*	1314	1646 (79.8)		822	1071 (76.8)		1204	1541 (78.2)		622	814 (76.4)	
Birthweight						0.03						0.063
*Normal/high*	1205	1566 (77.0)	0.350	826	1118 (73.8)		1394	1892 (73.7)	0.032	876	1202 (72.9)	
*Low*	98	120 (81.7)		68	80 (86.0)		89	106 (84.0)		84	102 (82.6)	
Breastfeeding						0.558						0.244
*Never breastfed*	50	63 (80.0)	0.877	21	32 (63.9)		7	9 (76.7)	0.278	49	67 (72.6)	
*Ever breastfed*	271	346 (78.2)		264	362 (73.0)		473	650 (72.8)		259	381 (67.8)	
*Still breastfeeding*	2248	2910 (77.3)		1342	1815 (73.9)		1808	2375 (76.1)		861	1150 (74.8)	
Dietary diversity score												0.952
*≥5*	NA	NA		NA	NA		1895	2489 (76.1)	0.087	1016	1390 (73.1)	
*<5*	NA	NA		NA	NA		393	545 (72.0)		152	208 (72.9)	
Stunting						0.439						0.516
*Normal*	1684	2214 (76.1)	<0.001	943	1272 (74.1)		1534	2052 (74.7)	0.346	802	1106 (72.5)	
*Stunted*	832	988 (84.2)		645	892 (72.3)		739	965 (76.6)		362	485 (74.5)	
Weight						0.803						0.116
*Underweight*	564	652 (86.6)	<0.001	267	373 (71.6)		335	417 (80.4)	0.078	164	204 (80.5)	
*Normal*	1913	2498 (76.6)		1302	1766 (73.7)		1917	2574 (74.5)		987	1373 (71.9)	
*Overweight*	38	52 (73.9)		19	25 (75.9)		32	39(81.4)		13	18 (75.5)	
Residence						0.009						0.075
*Urban*	450	604 (74.6)	0.147	344	433 (79.6)		597	816 (73.2)	0.146	336	433 (77.5)	
*Rural*	2119	2715 (78.1)		1285	1782 (72.1)		1691	2218 (76.2)		832	1165 (71.4)	
Insurance						0.645						<0.001
*Yes*	NA	NA		61	80 (73.5)		142	219 (64.9)	0.001	33	67 (48.8)	
*No*	NA	NA		1568	2133 (73.5)		2146	2815 (76.2)		1135	1531 (74.1)	
Children aged <5 years						0.133						0.240
*1*	837	1088 (76.9)	0.357	495	676 (73.2)		847	1131 (74.9)	<0.001	431	590 (73.1)	
*2*	1001	1299 (77.1)		667	937 (71.2)		834	1156 (72.1)		492	692 (71.1)	
*3*	474	620 (76.5)		292	379 (77.1)		384	477 (80.4)		141	192 (73.6)	
*>3*	256	312 (82.1)		175	222 (78.7)		223	269 (83.0)		85	103 (82.4)	
Mother’s age at first birth in years						0.587						0.223
*<15*	66	88 (75.1)	0.223	50	73 (68.3)		59	79 (75.1)	0.497	20	23 (86.6)	
*15–19*	1599	2065 (77.4)		967	1331 (72.7)		1347	1775 (75.9)		663	896 (74.0)	
*20–24*	754	986 (76.5)		529	708 (72.7)		732	969 (75.5)		390	539 (72.3)	
*≥25*	151	180 (83.9)		84	103 (81.6)		152	212 (71.6)		58	90 (64.3)	
Mother’s education level						0.081						0.689
*No education*	671	848 (79.1)	0.314	421	553 (76.1)		477	599 (79.7)	0.075	237	327 (72.5)	
*Primary*	1772	2317 (76.5)		1079	1502 (71.8)		1431	1911 (74.9)		677	920 (73.5)	
*Secondary*	102	123 (82.5)		122	152 (80.2)		357	494 (72.4)		248	339 (73.1)	
*Higher*	25	31 (78.7)					22	30 (75.5)		6	11 (54.4)	
Mother’s marital Status						0.038						0.182
*Married*	1982	2561 (77.4)	0.524	1262	1754 (71.9)		1368	1818 (75.2)	0.966	686	959 (71.5)	
*Living together*	249	335 (74.4)		101	124 (81.6)		512	684 (74.9)		268	370 (72.4)	
*Widowed/divorced/live apart*	181	226 (80.1)		163	202 (80.7)		223	294 (75.7)		105	132 (79.7)	
*Never married*	157	197 (79.7)		104	136 (76.5)		184	237 (77.8)		109	136 (79.8)	
Family size						0.297						0.820
*1–4*	654	849 (77.0)	0.671	406	543 (74.8)		550	776 (70.8)	<0.001	376	508 (74.0)	
*5–9*	1424	1848 (77.0)		880	1222 (72)		1248	1672 (74.6)		633	876 (72.2)	
*10+*	492	622 (79.0)		343	450 (76.2)		490	586 (83.7)		158	213 (74.2)	
Wealth index						0.017						0.532
*Poorest*	610	749 (81.5)	0.015	347	497 (69.8)		575	728 (79.0)	0.084	262	366 (71.5)	
*Poorer*	561	728 (77.1)		421	542 (77.6)		489	653 (74.9)		219	300 (73.0)	
*Middle*	544	719 (75.6)		324	465 (69.6)		437	569 (76.7)		217	310 (70.2)	
*Richer*	513	649 (79.1)		282	388 (72.8)		419	566 (74.0)		265	341 (77.5)	
*Richest*	341	474 (71.9)		256	323 (79.3)		368	517 (71.1)		205	281 (72.9)	
Household insecticide-treated net use						0.632						0.977
*Yes*	453	581 (77.9)	0.803	1075	1454 (73.9)		1303	1730 (75.3)	0.893	795	1088 (73.1)	
*No*	2116	2738 (77.3)		554	760 (72.9)		985	1304 (75.5)		373	510 (73.1)	

NA – not available

### Determinants of child anaemia in Tanzania

In the multivariable logistic regression model, the odds of child anaemia in 2022 did not differ significantly from those in 2015–2016 (adjusted odds ratio (aOR) = 0.955; 95% confidence interval (CI) = 0.782–1.166). Several individual and household characteristics were independently associated with anaemia. Boys were more likely to be anaemic than girls (aOR = 1.380; CI = 1.133–1.681), while underweight children faced 43.8% higher odds of anaemia compared to their non-underweight peers (aOR = 1.438; 95% CI = 1.032–2.005). Moreover, children with low birth weight bore a substantially higher burden of anaemia, with an estimated 71.3% increased risk compared to those with normal birth weight (aOR = 1.713; 95% CI = 1.076–2.727). At the household level, the absence of health insurance was associated with a 76.8% higher risk of child anaemia (aOR = 1.768; 95 CI = 1.247–2.508) (**Table 2**).

**Table 2:** The aORs of child anaemia (6–23 months) and its determinants in Tanzania

Variables	aOR (95% CI)	*P*-value
Phase		
*2015–2016*	ref	
*2022*	0.955 (0.782–1.166)	0.651
Age in months		
*6–11*	ref	
*12–23*	0.863 (0.700–1.065)	0.169
Sex		
*Female*	ref	
*Male*	1.380 (1.133–1.681)	0.001
Birthweight		
*Normal/high*	ref	
*Low*	1.713 (1.076–2.727)	0.023
Breastfeeding		
*Never breastfed*	ref	
*Ever breastfed*	0.523 (0.135–2.029)	0.348
*Still breastfeeding*	0.659 (0.172–2.522)	0.542
Dietary diversity score		
*≥5*	ref	
*<5*	0.925 (0.715–1.200)	0.555
Stunting		
*Normal*	ref	
*Stunted*	1.046 (0.842–1.300)	0.683
Weight		
*Normal*	ref	
*Underweight*	1.438 (1.032–2.005)	0.032
*Overweight*	1.738 (0.691–4.333)	0.241
Residence		
*Urban*	ref	
*Rural*	0.836 (0.626–1.117)	0.373
Insurance		
*Yes*	ref	
*No*	1.768 (0.626–1.117)	0.001
Number of children aged <5 years		
*1*	ref	
*2*	0.853 (0.681–1.067)	0.163
*3*	1.186 (0.826–1.701)	0.355
*>3*	1.172 (0.668–2.057)	0.580
Mother’s age in years at first birth		
*<15*	ref	
*15–19*	0.807 (0.375–1.736)	0.582
*20–24*	0.764 (0.349-1.674)	0.501
*≥25*	0.746 (0.330–1.677)	0.477
Mother’s education level		
*No education*	ref	
*Primary*	0.881 (0.629–1.234)	0.461
*Secondary*	0.942 (0.640–1.387)	0.763
*Higher*	1.321 (0.549–3.180)	0.534
Mother’s marital status		
*Married*	ref	
*Living together*	0.917 (0.720–1.167)	0.481
*Widowed/divorced/live apart*	1.215 (0.880–1.687)	0.244
*Never married*	1.240 (0.880–1.746)	0.218
Wealth index		
*Poorest*	ref	
*Poorer*	0.978 (0.721–1.327)	0.887
*Middle*	1.000 (0.709–1.410)	0.999
*Richer*	1.029 (0.720–1.470)	0.875
*Richest*	0.861 (0.556–1.333)	0.501
Family size		
*1–4*	ref	
*5–9*	1.010 (0.809–1.261)	0.929
*10+*	1.397 (0.932–2.093)	0.105
Household insecticide-treated net use		
*Yes*	ref	
*No*	0.915 (1.247–2.508)	0.373

aOR – adjusted odds ratio, CI – confidence interval, ref – reference

### Sensitivity analyses results

The results of the sensitivity analyses indicated that the main findings of this study were generally robust (Table S3–5 in the **Online Supplementary Document**). Low birth weight and child sex remained significant factors of anaemia across all sensitivity models. After multiple imputation for missing birthweight data, children from households with more than 10 members had significantly higher odds of anaemia by 46.9%, compared with those from households with one to four members (aOR = 1.469; CI = 1.069–2.017). When we applied cut-offs for moderate anaemia, we found that children surveyed in 2022 had 25.7% higher odds of anaemia than those surveyed in 2015–2016 (aOR = 1.257; 95% CI = 1.040–1.520).

## DISCUSSION

Based on multiple waves of TDHS data, we found that child anaemia in Tanzania did not decline as expected by 2022 and failed to achieve the projected 50.0% reduction. The prevalence remained statistically unchanged at 58.7% and 59.9% in 2015 and 2022, respectively, indicating stagnation in progress. The observed stagnation in the decline of child anaemia may be attributable to specific, vulnerable subgroups, particularly children aged <2 years. Alongside other associated factors such as underweight status, low birth weight is a prominent risk factor for elevated anaemia in these age groups.

### The heavy burden of low-birth-weight children and anaemia vulnerability in the first 1000 days

The first 1000 days are a critical window for children’s growth and development [22]. Our findings highlight the heavy burden of anaemia among low birth weight children, underscoring the significance of the gestation within this period. Low birth weight is commonly attributed to adverse maternal conditions during pregnancy, including malnutrition, anaemia, infections, inadequate antenatal care, and pregnancy-related complications [15,23-25]. In Tanzania, studies have found that maternal anaemia and other nutrient deficiencies remain widespread among pregnant women, hindering the nutrient supply necessary for optimal intrauterine growth [26,27]. Evidence from LMICs indicates that micronutrient supplementation for pregnant women can significantly decrease the occurrence of low birth weight, small-for-gestational-age infants, and premature labour [28,29]. Strengthening maternal nutrition is therefore critical to breaking the intergenerational cycle of undernutrition and child anaemia.

Children aged <2 years in Tanzania face a markedly higher anaemia prevalence than older children, reflecting the unique nutritional demands of this developmental stage. Based on existing experience, infants’ rapid growth, coupled with inadequate access to micronutrient supplementation or fortified complementary foods, heightens the risk of anaemia and compromises both physical and cognitive development [30,31]. Tailored interventions should be advocated for children aged <2 years, including improved complementary feeding and timely micronutrient supplementation [32,33]. It is challenging for LMICs to achieve substantial improvements in feeding practices, and nutrient supplementation can be considered as an intervention. Programs such as the Ying Yang Bao initiative in China have demonstrated tangible success in resource-limited settings, providing valuable experience in reducing infant anaemia [34].

However, the observed association between low birth weight and child anaemia should be interpreted with caution due to potential measurement errors in birthweight, especially for children not weighed at birth, where values may rely on maternal recall.

### Socioeconomic and biological determinants of child anaemia

Children from households without health insurance experience a heavier burden of anaemia, reflecting the influence of socioeconomic disparities on child health. Lack of health insurance reduces access to essential antenatal services, which are vital for reducing both maternal and neonatal risks [35,36]. Moreover, the absence of insurance often indicates a household’s low socioeconomic status, which is an indicator of overall poverty [35]. This socioeconomic disadvantage constrains children’s dietary diversity, caregivers’ healthcare-seeking behaviours, and household resilience against nutritional deficiencies. These combined challenges contribute to intergenerational cycles of malnutrition and anaemia, particularly in resource-limited settings such as Tanzania.

Beyond socioeconomic disparities, biological differences also contribute to unequal anaemia risks. Boys were observed to bear a higher anaemia burden than girls. This disparity may be attributed to genetic factors and to the observation that male infants typically have higher iron requirements than female infants [37,38].

### Regional disparities as a contributing factor to the stagnation of child anaemia reduction in Tanzania

In this study, we found significant regional variation and variability in child anaemia across Tanzania. The prevalence of anaemia among children and its improvement were relatively poor, especially in the eastern coastal regions. They rely heavily on marine food sources. However, in recent years, these regions have become increasingly vulnerable to food insecurity due to ocean warming, shifting currents, and other climate-related changes in the marine environment, compounded by the effects of overfishing [39,40]. In island areas in particular, the diversity of major food sources is even more limited, owing to the scarcity of arable land and the lack of alternative income-generating activities [41]. These causes contribute to the nutritional problems of children in the area.

The burden of child anaemia remains high in Tanzania’s main food-producing regions. This issue may partly be attributed to farmers in central and southern Tanzania prioritising the sale of their crops over household consumption, thereby reducing local food availability. The predominantly monotonous diets in these regions heighten the risk of micronutrient deficiencies among children. Furthermore, food contamination continues to pose a significant threat. Cereals, particularly maize grown in areas such as Mbeya, Morogoro, and Mtwara, are especially susceptible to aflatoxin contamination, which has been linked to adverse health outcomes in children, including stunting and anaemia [42].

### Limitations

This study has several limitations. First, we relied on cross-sectional data from the TDHS, which restricts causal inference between anaemia and its associated factors. Moreover, self-reported information may be subject to social desirability bias given the nature of the data collection. Second, the study was constrained by missing data on certain variables, particularly birth weight and dietary diversity. Although we applied multiple imputation to birth weight to reduce potential bias, some residual uncertainty may remain. For the dietary diversity score, we coded missing responses as non-consumption in accordance with DHS guidelines, potentially leading to an underestimation of actual dietary diversity. Furthermore, we did not include unmeasured confounders such as infections and hemoglobinopathies due to data limitations, which could bias the observed associations for nutritional and socioeconomic status indicators. Finally, despite the use of standardised field protocols, measurement bias may exist across survey rounds, particularly due to differences in haemoglobin testing devices.

## CONCLUSIONS

We found that child anaemia in Tanzania has stagnated since 2015, failing to achieve the projected 50.0% reduction by 2022. The burden is disproportionately concentrated among children born with low birth weight and those aged <2 years, underscoring the critical importance of maternal and early childhood nutrition within the first 1000 days. Moreover, substantial regional disparities persist, with some coastal and high food-production areas exhibiting higher anaemia prevalence, indicating uneven progress across the country. These findings highlight the need for intensified, region-specific strategies targeting the most vulnerable subgroups to accelerate anaemia reduction in LMICs.

## Additional material


Online Supplementary Document
