# Supplementary material for: The stagnation of child anaemia (6–23 months) in Tanzania from 2004 to 2022: a missed opportunity during the ‘first 1000 days’
Source: J Glob Health. 2026 Feb 13;16:04033. doi: 10.7189/jogh.16.04033 (PMC12900548; doi:10.7189/jogh.16.04033)
Supplement: Online Supplementary Document [file jogh-16-04033-s001.pdf]

Supplement to: Wang Y, Sunguya B, Na M, Ally MS, Huang J. The stagnation of child anaemia (6–23 months) in Tanzania from 2004–22: a missed opportunity during the 'first 1000 days'. J Glob Health. 2026;16:04033.

Table S1 Missing data for covariates

Variable	2004/2005		2010		2015/2016		2022	
	Total, N	Missing, n(%)	Total, N	Missing, n(%)	Total, N	Missing, n(%)	Total, N	Missing, n(%)
Birthweight	3319	1633(49.20)	2215	1017(45.9)	3034	1036(34.15)	1598	294(18.40)
Stunting	3319	117(3.53)	2215	51(2.30)	3034	17(0.56)	1598	7(0.44)
weight	3319	117(3.53)	2215	51(2.30)	3034	4(0.13)	1598	4(0.25)

Table S2 Multicollinearity test for covariates based on generalized variance inflation factor

variable	GVIF	Df	GVIF^(1/(2*Df))
Phase	1.0514	1	1.0254
Age	1.1791	1	1.0858
Sex	1.0263	1	1.0131
Birthweight	1.0331	1	1.0164
Breastfeeding	1.2013	2	1.0469
Dietary diversity score	1.1158	1	1.0563
Stunting	1.1691	1	1.0812
Weight	1.1123	1	1.0547
Residence	1.6211	1	1.2732
Insurance	1.1037	1	1.0506
Number of children under 5	1.6050	3	1.0820
Mother's age at first birth	1.3102	3	1.0461
Education level (mother)	1.6430	3	1.0863
Mother's Marital Status	1.1333	3	1.0211
Wealth index	2.1836	4	1.1025
Family size	1.5881	2	1.1225
Household insecticide-treated net use	1.0299	1	1.0148

Table S3 The AORs of child anemia(6-23months) and its determinants in Tanzania by multiple imputation

Variable	AOR	95%CI	P
**Phase**			
2015/2016	1		
2022	0.903	0.754-1.081	0.267
**Individual Characteristics**			
**Age**			
6—11	1		
12—23	0.841	0.699-1.012	0.067
**Sex**			
female	1		
male	1.365	1.154-1.614	0.000***
**Birthweight**			
Normal/high	1		
Low	1.717	1.096-2.688	0.018*
**Breastfeeding**			
never breastfeeding	1		
ever Breastfeeding	0.914	0.340-2.459	0.858
still Breastfeeding	1.135	0.426-3.029	0.799
**Dietary diversity score**			
>=5	1		
<5	0.931	0.742-1.168	0.536
**Stunting**			
normal	1		
stunted	0.998	0.827-1.205	0.985
**Weight**			
normal	1		
underweight	1.400	1.063-1.844	0.016*
overweight	1.340	0.591-3.041	0.483
**Household characteristics**			
**Residence**			
Urban	1		
Rural	0.886	0.685-1.147	0.359
**Insurance**			
yes	1		
no	1.831	1.327-2.527	0.000***
**Number of children under 5**			
1	1		
2	0.841	0.688-1.029	0.093
3	1.049	0.795-1.385	0.733
>3	1.171	0.766-1.791	0.465
**Mother's age at first birth**			
<15	1		

15—19	0.967	0.582-1.609	0.898
20—24	0.959	0.564-1.630	0.877
≥25	0.844	0.474-1.501	0.562
**Education level (mother)**			
No education	1		
Primary	0.870	0.691-1.097	0.239
Secondary	0.882	0.644-1.207	0.432
Higher	1.237	0.546-2.806	0.609
**Mother's Marital Status**			
Married	1		
Living together	0.972	0.788-1.199	0.794
Widowed/Divorced/Live Apart	1.107	0.829-1.478	0.49
Never married	1.242	0.905-1.705	0.180
**Wealth index**			
Poorest	1		
poorer	0.929	0.737-1.171	0.531
Middle	0.949	0.731-1.233	0.695
Richer	1.019	0.738-1.406	0.911
Richest	0.904	0.615-1.329	0.608
**Family size**			
1—4	1		
5—9	1.087	0.895-1.320	0.401
10+	1.469	1.069-2.017	0.018*
**Household insecticide-treated net use**			
yes	1		
no	1.012	0.816-1.256	0.913

*<0.05, **<0.01, ***<0.001

Table S4 The AORs of child anemia(6-23months) and its determinants in Tanzania excluding weight

Variable	AOR	95%CI	P
**Phase**			
2015/2016	1		
2022	0.947	0.776-1.156	0.593
**Individual Characteristics**			
**Age**			
6—11	1		
12—23	0.855	0.692-1.056	0.145
**Sex**			
female	1		
male	1.406	1.156-1.709	0.001**
**Birthweight**			
Normal/high	1		
Low	1.773	1.119-2.811	0.015*
**Breastfeeding**			
never breastfeeding	1		
ever Breastfeeding	0.512	0.132-1.985	0.333
still Breastfeeding	0.650	0.170-2.481	0.528
**Dietary diversity score**			
>=5	1		
<5	0.922	0.713-1.193	0.536
**Stunting**			
normal	1		
stunted	1.134	0.921-1.395	0.235
**Household characteristics**			
**Residence**			
Urban	1		
Rural	0.832	0.624-1.110	0.211
**Insurance**			
yes	1		
no	1.796	1.268-2.544	0.001**
**Number of children under 5**			
1	1		
2	0.854	0.683-1.067	0.165
3	1.199	0.835-1.721	0.325
>3	1.178	0.673-2.062	0.566
**Mother's age at first birth**			
<15	1		
15—19	0.768	0.358-1.648	0.497
20—24	0.734	0.336-1.603	0.437
≥25	0.719	0.321-1.613	0.423
**Education level (mother)**			
No education	1		

Primary	0.890	0.635-1.247	0.498
Secondary	0.948	0.644-1.394	0.785
Higher	1.306	0.542-3.142	0.551
**Mother's Marital Status**			
Married	1		
Living together	0.913	0.716-1.163	0.459
Widowed/Divorced/Live Apart	1.203	0.867-1.668	0.268
Never married	1.232	0.877-1.731	0.229
**Wealth index**			
Poorest	1		
poorer	0.977	0.721-1.326	0.883
Middle	1.007	0.713-1.422	0.967
Richer	1.021	0.714-1.461	0.908
Richest	0.852	0.550-1.320	0.472
**Family size**			
1—4	1		
5—9	1.015	0.814-1.267	0.893
10+	1.373	0.917-2.055	0.124
**Household insecticide-treated net use**			
yes	1		
no	0.909	0.748-1.104	0.335

*<0.05, **<0.01, ***<0.001

Table S5 The AORs of child anemia(6-23months) and its determinants in Tanzania using alternative cut-offs for moderate anemia

Variable	AOR	95%CI	P
**Phase**			
2015/2016	1		
2022	1.257	1.040-1.520	0.018*
**Individual Characteristics**			
**Age**			
6—11	1		
12—23	0.841	0.694-1.020	0.079
**Sex**			
female	1		
male	1.436	1.206-1.709	0.000***
**Birthweight**			
Normal/high	1		
Low	2.206	1.555-3.129	0.000***
**Breastfeeding**			
never breastfeeding	1		
ever Breastfeeding	0.642	0.279-1.481	0.299
still Breastfeeding	0.774	0.338-1.774	0.545
**Dietary diversity score**			
>=5	1		
<5	0.911	0.717-1.157	0.444
**Stunting**			
normal	1		
stunted	1.105	0.908-1.345	0.317
**Weight**			
normal	1		
underweight	1.264	0.961-1.662	0.094
overweight	1.244	0.623-2.483	0.536
**Household characteristics**			
**Residence**			
Urban	1		
Rural	0.824	0.627-1.082	0.164
**Insurance**			
yes	1		
no	1.228	0.862-1.750	0.254
**Number of children under 5**			
1	1		
2	0.846	0.694-1.031	0.097
3	1.102	0.803-1.514	0.547
>3	1.352	0.854-2.141	0.198
**Mother's age at first birth**			

<15	1		
15—19	0.945	0.488-1.830	0.867
20—24	0.797	0.409-1.554	0.504
≥25	0.714	0.339-1.504	0.375
**Education level (mother)**			
No education	1		
Primary	0.800	0.606-1.054	0.113
Secondary	0.801	0.583-1.101	0.171
Higher	1.155	0.482-2.769	0.747
**Mother's Marital Status**			
Married	1		
Living together	0.996	0.790-1.256	0.976
Widowed/Divorced/Live Apart	1.082	0.823-1.421	0.573
Never married	1.037	0.756-1.422	0.821
**Wealth index**			
Poorest	1		
poorer	0.920	0.682-1.240	0.584
Middle	1.031	0.740-1.436	0.858
Richer	1.089	0.788-1.506	0.604
Richest	0.958	0.644-1.426	0.834
**Family size**			
1—4	1		
5—9	0.834	0.677-1.027	0.088
10+	0.999	0.699-1.428	0.997
**Household insecticide-treated net use**			
yes	1		
no	0.948	0.795-1.130	0.552

*<0.05, **<0.01, ***<0.001
